# Mesenchymal Stromal Cell Uses for Acute Kidney Injury—Current Available Data and Future Perspectives: A Mini-Review

**DOI:** 10.3389/fimmu.2020.01369

**Published:** 2020-07-21

**Authors:** Shani Zilberman-Itskovich, Shai Efrati

**Affiliations:** ^1^Nephrology Division, Assaf-Harofeh (Shamir) Medical Center, Be'er Ya'akov, Israel; ^2^Sackler School of Medicine, Tel-Aviv University, Tel-Aviv, Israel

**Keywords:** acute renal failure, AKI, mesenchymal stem cells, MSC, immune response

## Abstract

There is growing evidence concerning the potential use of mesenchymal stromal cells (MSCs) for different tissue injuries. Initially, the intended physiological use of MSCs was due to their ability to differentiate and replace damaged cells. However, MSCs have multiple effects, including being able to significantly modulate immunological responses. MSCs are currently being tested for neurodegenerative diseases, graft vs. host disease, kidney injury, and other chronic unremitting tissue damage. Using MSCs in acute tissue damage is only now being studied. Acute kidney injury (AKI) is a common cause of morbidity and mortality. After the primary insult, overactivation of the immune system culminates in additional secondary potentially permanent kidney damage. MSCs have the potential to ameliorate the secondary damage, and recent studies have shed important light on their mechanisms of action. This article summarizes the basics of MSCs therapy, the newly discovered mechanisms of action, and their potential application in the setting of AKI.

## Introduction

Acute kidney injury (AKI) is a syndrome of rapid renal function deterioration over a period of hours or days (1). AKI is a common cause of morbidity and mortality, complicating 20% of hospitalized patients, half of them needing renal replacement therapy (2). This severe form of AKI is related to a 50% increase in mortality among other devastating long-term consequences, including end-stage renal disease (ESRD), and dialysis dependence (2, 3).

The etiologies of AKI are varied and are usually divided into pre-, intra-, and post-renal categories (4). The most common category is prerenal, secondary to hypoperfusion of the kidney. Hypoperfusion of the kidney can be caused by reduced effective blood volume secondary to hemorrhage, cardiac insufficiency, and/or third spacing of fluids (4). Intrarenal AKI is caused by intrinsic injury to the renal tissue, with the most common injury being acute tubular necrosis secondary to ischemia–reperfusion injury (IRI) (5–7). Other intrinsic renal causes include glomerular pathologies such as glomerulonephritis and interstitial and vascular injury (4). Postrenal AKI occurs from obstruction of the ureters, bladder outlet, or urethra (4). Irrespective of the primary cause, an intrarenal inflammatory cascade is activated following the acute kidney injury, which if not quickly controlled, culminates in additional renal damage and irreversible renal fibrosis (detailed below).

In the last decade, cumulative evidence has shown the significant role that overactivated immune responses play in the development of AKI (8). This understanding paved the way to new therapeutic strategies for this relatively common and life-threatening acute kidney condition. Unfortunately, despite the progress in our understanding of AKI biology, treatment options for AKI in the daily clinical setting are still limited (1, 3, 5). While dialysis can be relatively effective in handling the hazardous electrolytes and volume complications as a supportive therapy, there is a need for a treatment that can counteract the pathological cascade that can culminate in irreversible loss of renal tissue (1, 5).

## The Immune Response to Acute Kidney Injury

The immune system plays a crucial role in AKI mechanisms with involvement of both the innate and adaptive immune system branches (9). Regarding the innate immune system, cytokines serve as major mediators with both increased production of cytokines and reduced clearance being reported during AKI (10). Interleukin (IL)-6, IL-8, and tumor necrosis factor (TNF)-α are usually elevated and are related to endothelial dysfunction and tubular injury (11). Conversely, IL-10 has an ameliorating effect by promoting immune tolerance (12). In the AKI setting, growth factors also play a role by regulating inflammation and programmed cell death. When administrated early after the acute insult, growth factors such as the epidermal growth factor (EGF), insulin-like growth factor (IGF), and fibroblast growth factor (FGF) can promote renal repair and renal function restoration in animal models (13).

The complement system, a part of the innate immune system, also has an important role in the pathogenesis of renal injury and is involved in glomerular, tubulointerstitial, and vascular kidney injuries (14). The final common pathway of the complement system is the membrane attack complex that induces direct cellular damage and causes activation and migration of neutrophils, which further amplifies the injury (15). Suppressing the complement system in AKI has shown promising results in preclinical studies (16).

The cellular response to AKI includes both pro- and anti-inflammatory characteristics. Dendritic cells, monocytes/macrophages, neutrophils, T lymphocytes, and B lymphocytes are all involved in AKI and can be detected as early as 1 h after the acute insult (17). The involvement of these cells can directly and indirectly induce apoptosis of the renal tubular cells (17). Neutrophils recruited to the injured kidney cause vascular congestion that, together with the secreted cytotoxic compounds, including reactive oxygen species, further exacerbate tissue damage (11). M1 macrophages release chemokines, proinflammatory cytokines, and inducible nitric oxide synthase, which form peroxynitrites. These peroxynitrites have a vasoconstrictive effect, which can aggravate the ischemic and inflammatory damage (11). Lymphocytes enhance AKI by releasing IL-17, a proinflammatory cytokine that also increases vascular permeability (11, 17, 18). In contrast, M2 macrophages and regulatory T cells are essential for suppressing the overactivated inflammatory response and for regenerating damaged renal tissue and are detected while recovering from the acute insult (9).

The relation between the different arms of the immune system can either escalate or downgrade the final injury (11, 15). To steer the cells and factors toward a less devasting rout, new treatments are being investigated including the use of stem-cell therapy.

## Mesenchymal Stromal Cells

Mesenchymal stromal cells (MSCs) are fibroblast-like multipotent cells that can differentiate into mesodermal-line cells including adipocytes, chondroblasts, osteoblasts, and renal tubular cells (19, 20). These cells exhibit self-renewal properties, with a potential to replace damaged cells (21). MSCs are defined by three main characteristics: (1) plastic adherent when maintained in standard culture conditions; (2) expression of CD105, CD73, and CD90, with no expression of other CDs that are not mesenchyme related [including CD45, CD34, CD14, or CD11b, CD79-α, or CD19 and human leukocyte antigen (HLA)-DR surface molecules]; and (3) the ability to differentiate into a mature mesoderm related cell line *in vitro* (22). Unlike embryonic stem cells, MSCs are found in many organs even in adults (20, 22, 23).

In the past two decades, MSCs from different origins are being used in different clinical trial settings (24). For example, bone-marrow-derived MSCs are used in children to treat graft-vs.-host disease, autologous marrow MSCs for heart disease (23), and both bone-marrow and adipose-derived MSCs are used in Crohn's-related enterocutaneous fistular disease (25). In the neurodegenerative field, MSCs are being studied in amyotrophic lateral sclerosis, multiple system atrophy, Parkinson's disease, Alzheimer's disease, and multiple sclerosis. While animal studies have been promising, clinical studies have demonstrated conflicting results (26, 27). The encouraging results obtained in the field of degenerative diseases can be related, among others, to the effect that MSCs have on the immune factors in these disease settings (26, 27).

## The Biology of Mesenchymal Stromal Cells

MSCs can affect and be affected by other cells through different immune mediators. Cytokines, chemokines, and transcription factors can influence the differentiation of MSCs. Expression in MSCs of specific transcription factors, including Runx2, Sox9, PPARγ, MyoD, GATA4, and GATA6, promote their differentiation into specific cell lineage (20).

The primary rationale for using MSCs to rejuvenate damaged tissue was initially related to their ability to differentiate into the damaged tissue-related cells. Following IRI, MSCs migrate to the injured site and alleviate the damage (21). Studies have demonstrated that MSCs have beneficial effects even at very early stages after their migration, before any differentiation and proliferation can be expected (28). This observation has led to the understanding that the MSC's early beneficial effects are related to their paracrine activity in the surrounding tissue (29, 30). Recent studies have demonstrated that MSCs can induce both local and remote anti-inflammatory effects (31). The immunomodulatory effects of MSCs are broad and cover much of the innate and adaptive immune systems (19). For example, MSCs can secrete factors such as insulin-like growth factor-1 (IGF-1), vascular endothelial growth factor (VEGF), angiopoietin 1, keratinocyte growth factor, and macrophage inflammatory protein 1α. These broad signaling factors are capable of promoting cell proliferation, angiogenesis, and wound healing (30). Paracrine or extracellular vesicle-delivered growth factors, such as hepatocyte growth factor (HGF) or VEGF, represent additional mechanisms by which MSCs exert therapeutic effects on renal injury (13).

MSCs can present both pro- and anti-inflammatory profiles. These different phenotypes are related to their ability to sense the environment and respond to changes in the tissue. The effect is induced by activation of different macrophage populations (19). Macrophages are divided to two main groups: M1 and M2 macrophages. M1 macrophages are considered proinflammatory cells and secrete proinflammatory cytokines including IL-1, IL-6, TNF-α, and interferon-γ. M2 macrophages are anti-inflammatory cells that secret anti-inflammatory cytokines such as IL-10 and transforming growth factor (TGF)-β1 (19, 32, 33). Thus, MSCs can induce differentiation of monocytes to one of the macrophage phenotype groups according to the inflammatory status of the damaged tissue (19). MSCs can also affect T-cell activation and differentiation toward T-regulatory cells that have anti-inflammatory properties (34). In addition to the paracrine effects on the immune system, MSCs can transfer mitochondria into the damaged cells, enabling better energy utilization, and restoration of the adenosine triphosphate (ATP) supply, thus promoting cellular recuperation (34). MSCs might also assist in preserving tubular mitochondria, thus preserving the functionality of these cells (35). By improving oxygen metabolism and energy utilization, MSCs reduce the oxidative stress and induce antioxidant activity (36).

To conclude, MSCs can promote tissue regeneration even before differentiating into the damaged cell line of the injured tissue. This influence is related to their early multifaceted paracrine effects.

## Treatment With Mesenchymal Stromal Cells in Acute Kidney Injury

**Figure 1:**
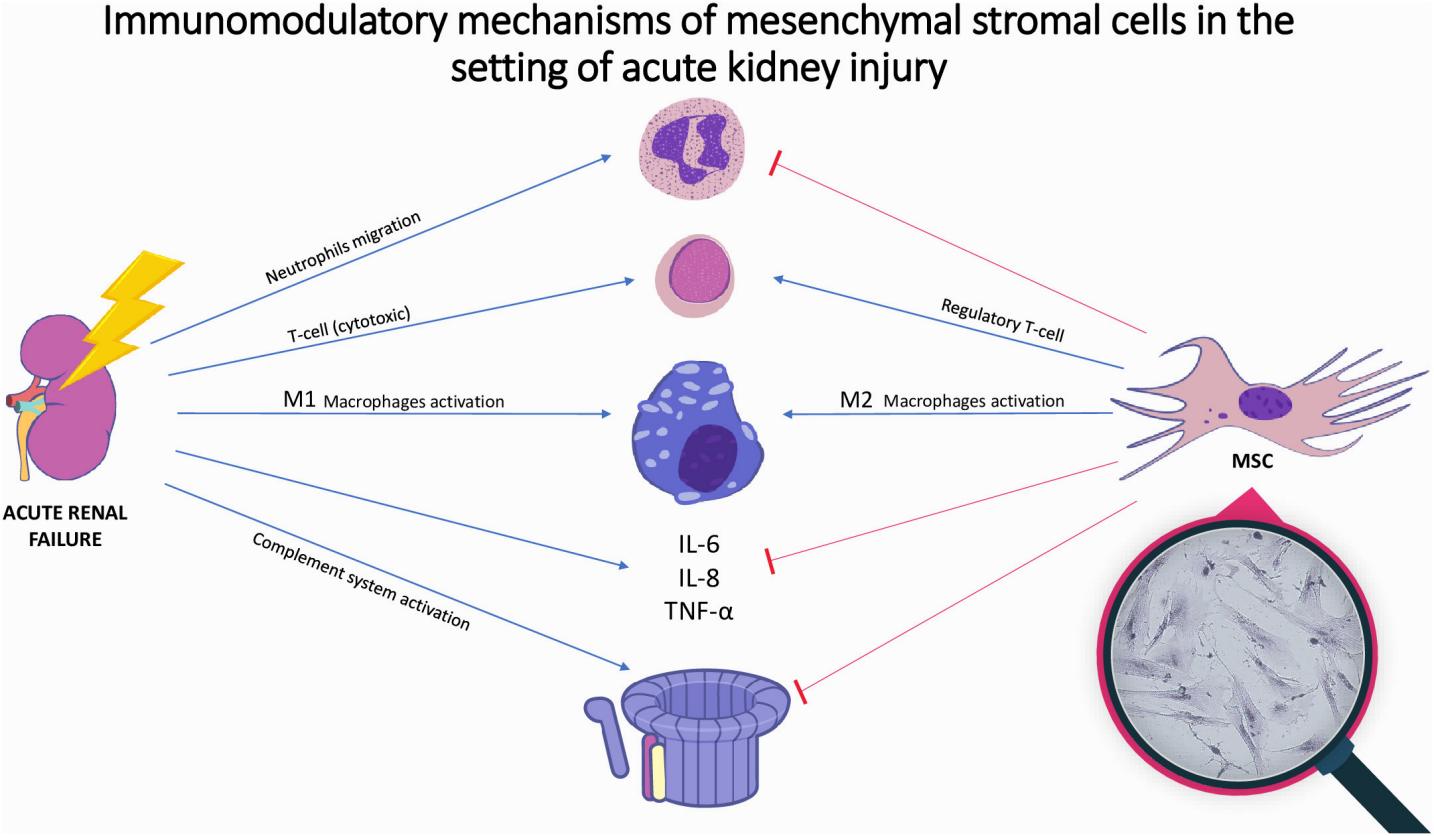
Immunomodulatory mechanisms of mesenchymal stromal cells in the setting of acute kidney injury. Illustration of the immune mechanisms of acute kidney injury and the immunomodulatory effect of mesenchymal stromal cells. Acute kidney injury is accompanied by increase in inflammatory cytokines, complement activation and immune cell activation. Mesenchymal stromal cells inhibit cytokines release, complement system activation, and neutrophils migration, while promoting M2-anti-inflammatory macrophages and regulatory T-cells proliferation; In magnifying glass: microscopic picture of mesenchymal stromal cells-placenta origin; MSC, mesenchymal stromal cell; IL, Interleukin; TNF, tumor necrosis factor.

In the setting of AKI, MSCs promote protective effects on the injured kidney and ameliorate tissue damage (34, 36). The beneficial effects of MSCs are noticeable early after their injection and can be attributed to the following paracrine related mechanisms (Table 1 and Figure 1):

- An increase in the M2 macrophage CD68/CD163 population. As discussed, these M2 macrophages have anti-inflammatory and proregenerative properties (28, 32).
- A shift from the proinflammatory cytokines TNF-α, and IL-1β to the anti-inflammatory cytokine IL-10 with a favorable expression of the homing adhesion molecules intracellular adhesion molecules (ICAM-1) and vascular cell adhesion molecule 1 (VCAM-1) (50).
- An inhibitory effect on the complement system's overactivation and the related cellular damage generated by the membrane attack complex (28).
- Release of proangiogenesis growth factors such as VEGF, and proliferative growth factors (IGF, EGF) that promote cellular repair and promote cell regeneration (13, 51).
- Exosomes—one of the most exciting discoveries in intercellular communication. Exosomes are membrane-bound extracellular vesicles that are produced by most eukaryotic cells. Their size is about 30–120 nm in diameter (around the size of lipoproteins) and contain various molecular constituents of their cell of origin, including proteins, mRNA, and miRNA or double-stranded DNA (52, 53). Recent studies have demonstrated that administration of MSCs-derived exosomes can ameliorate the expected renal damage in the setting of AKI (52).
- Epigenetic effects—a shift in gene expression. Xie et al. (54) demonstrated that overexpression of the Klotho gene, which regulates apoptosis, can reinforce the protective effect of MSCs in the setting of AKI. Chen et al. demonstrated that the protective effect of MSCs in the setting of AKI can be related to TNF-inducible gene 6 protein expression. This protein, in addition to its anti-inflammatory effect, can promote renal tubular epithelial cell proliferation (55).

**Table 1:** Immunomodulatory mechanisms of mesenchymal stromal cells in the setting of acute kidney injury.

**Immune system component**	**Mechanism**	**References**
Complement system	Amelioration of complement system activation	(28, 37)
Cytokines	Downregulation of proinflammatory cytokines: IL-1β, IL-6, IL-17, TNF-α, INF-γ, TGF-β	(28, 38–43)
	Upregulation of anti-inflammatory cytokines: IL-10, IL-4, bFGF, and TGF-α	(28, 38, 39, 43–45)
Macrophages	Proliferation and migration of the M2 macrophage population	(28, 43, 46)
	Inhibition of macrophage infiltration	(45)
T-cells	Inhibition of T-cell infiltration	(43, 45)
	Differentiation to T-cell regulatory cells	(40, 43, 47, 48)
Neutrophils	Inhibition of neutrophils infiltration	(43, 49)

*IL, interleukin; TNF, tumor necrosis factor; INF, interferon; TGF, transforming growth factor; bFGF, basic fibroblast growth factor*.

While there is a growing body of knowledge in preclinical studies, the available clinical data on MSCs in AKI is still scarce. A recent study using MSCs in postcardiac surgery patients did not show beneficial effects regarding postsurgery AKI (56). This unfortunate result can be attributed to time of MSCs administration. The optimal results are obtained when MSCs are administered closest to the initiation of IRI (50). The detection of AKI, based on commonly used blood markers in humans (serum creatinine and urea), is usually late, after AKI and tubular necrosis are well-established (7). In this scenario, when the damage is already well-established, the potential immunological benefits of MSCs are probably negligible. In addition, the MSCs themselves might be injured by an overactivated complement system (57).

In addition to AKI, there is growing evidence of MSC benefits in the setting of chronic kidney disease (CKD). Even though the clinical studies done so far included relatively small numbers of patients, the evidence looks promising regarding the ability of MSCs to prevent the expected kidney function deterioration over time (58–60). In patients suffering from chronic diabetic nephropathy, allogeneic transplantation of MSCs demonstrated renal function improvements compared to placebo (60). The effect can be attributed to the paracrine secretion of VEGF and IGF-1 needed for angiogensis and tissue regeneration, and to the anti-inflammatory effect that controls the overactivated immune response that accompanies most CKD before significant sclerosis develops (59, 61).

One of the relevant clinical settings where MSCs potentially have beneficial effects is in postrenal transplantation patients. In the immediate posttransplantation period, IRI is one of the main reasons for AKI (62). Thanks to the above-discussed immunomodulating effects of MSCs, there are promising results in preclinical trials, and clinical studies are currently ongoing (63).

## Current Available MSC Safety Data

Several safety concerns are related to the use of MSCs in clinical settings. The first concern is related to the administration technique. When the cells are administered intravenously (IV), most of the cells are found within the lungs (28, 64). When the lung capillaries are blocked with these cells, ventilation, and respiratory difficulties ensue. Therefore, higher dosage with high concentration of MSCs should be avoided. The second concern is related to exposing the immune system to foreign cells when administering donor cells. Luckily, MSCs do not stimulate an intense immune response, since they only express the HLA-DR but lack other HLA typings (29). In a CKD trial, none of the patients developed persistent donor-specific anti-HLA antibodies (60). In particular, fetal MSCs have very low immunogenicity by nature and can be used to overcome this potential barrier (36). The last concern is related to the proliferation and differentiation of pluripotent cells injected to a living body, with their potential of being transformed into malignant cells. This concern is probably irrelevant, since stromal cells need a special environment and signaling factors to act as stem cells and differentiate, and usually do not survive after administration (20, 64). In any case, to address this scenario, more research with long-term follow-up is needed.

Even though clinical trials with long-term follow-up are still lacking, some preclinical trials have addressed the safety issues. Till now, no serious adverse effects were reported in either preclinical (65, 66) or clinical studies (21, 56, 60, 67).

## Summary and Future Perspectives

The ongoing cumulative data on the beneficial physiological effects of MSCs open new treatment opportunities for diseases that are currently being managed with only supportively therapy. While other types of stem cells, such as hematopoietic stem cells, are used in the clinical practice, the clinical data on MSCs is still scarce. In the setting of AKI, MSCs by way of their paracrine effects, can modulate the hazardous results of an overactivated inflammatory response. MSCs hold hope for future novel therapies, and a better understanding of the immune-biological effects of these cells will enable development of new treatment strategies.

## Author Contributions

SZ-I and SE conducted a review of articles and wrote the manuscript. Both authors contributed to the article and approved the submitted version.

## Conflict of Interest

The authors declare that the research was conducted in the absence of any commercial or financial relationships that could be construed as a potential conflict of interest.
